# An integrated behavioral neuroscience and educational psychology curriculum enhances medical interns’ engagement, resilience, and clinical performance in Saudi Arabia

**DOI:** 10.3389/fmed.2026.1743855

**Published:** 2026-03-06

**Authors:** Marwa M. Fawzi, Khaled A. Shahat, Nevein M. Abdelhady, Ayman A. Refai, Shaimaa M. Hassan, Zeinab A. Awadallah, Rana Hesham, Nour M. Laissy, Hanya Gaber, Rana A. Alduraywish, Tayseer Mansour

**Affiliations:** 1 Department of Basic Medical Sciences, Al-Rayan National College of Medicine, Al-Rayan National Colleges, Madina, Saudi Arabia; 2 Department of Forensic Medicine and Clinical Toxicology, Faculty of Medicine, Ain Shams University, Cairo, Egypt; 3 Department of Women and Child Health, College of Medicine, Taibah University, Madinah, Saudi Arabia; 4 Al-Rayan National College of Medicine, Al-Rayan National Colleges, Madina, Saudi Arabia; 5 Department of Clinical Pharmacy, Al-Rayan National College of Health Sciences, Al-Rayan National Colleges, Madina, Saudi Arabia; 6 Department of Anatomy and Physiology, College of Medicine, Imam Mohammad Ibn Saud Islamic University (IMSIU), Riyadh, Saudi Arabia; 7 Department of Histology and Cell Biology, Faculty of Medicine, Menoufia University, Shebeen El-Kom, Egypt; 8 Department of Histology, General Medicine Practice Program, Batterjee Medical College, Aseer, Saudi Arabia; 9 Department of Anatomy, College of Medicine, Taibah University, Madina, Saudi Arabia; 10 Department of Histology and Cell Biology, Faculty of Medicine, Zagazig University, Zagazig, Egypt; 11 Faculty of Medicine, Ain Shams University, Cairo, Egypt; 12 Al-Rayan National College of Medicine, Al-Rayan National Colleges, Madina, Saudi Arabia; 13 The Behman Hospital, Cairo, Egypt; 14 Department of Medical Education, Faculty of Medicine, Suez Canal University, Ismailia, Egypt; 15 Department of Family and Community Medicine and Medical Education, College of Medicine, Taibah University, Madinah, Saudi Arabia

**Keywords:** clinical competency, educational psychology, medical internship, neurodidactics, resilience training, retrieval practice, Saudi Arabia

## Abstract

**Background:**

The medical internship is a critical period of intense learning and professional development. During this time, medical graduates must acquire and apply vast amounts of knowledge under significant cognitive and emotional strain. Novel methods, such as integrating behavioral neuroscience, which examines how the brain enables learning and memory, manages emotions, and guides decision-making, with educational psychology, which explores how people are motivated, regulate their own learning, and think about their thinking, may support a more effective, brain-aligned, and sustainable training environment.

**Aim:**

This study assessed the feasibility and efficacy of a curriculum integrating neurodidactic and psychological techniques to improve resilience and clinical performance among Saudi medical interns.

**Methods:**

This study used a pragmatic, quasi-experimental, single-cohort longitudinal design with pre- and post-intervention assessments. The research team co-developed a curriculum comprising a series of educational modules that incorporated evidence-based learning strategies, including retrieval practice, spaced repetition, and interleaving. The curriculum also featured multimedia cognitive-load design, metacognitive coaching, mindfulness, reappraisal drills, and team communication simulations. One hundred participants completed the study. To evaluate the intervention’s impact, we used validated Arabic-language surveys to assess cognitive engagement, emotional resilience, and perceived stress. Faculty members rated clinical competencies using workplace-based assessments (mini-CEX), and focus groups were conducted to gather qualitative feedback.

**Results:**

The implementation yielded significant positive outcomes, with notable elevations in cognitive engagement (+12.4, *p* < 0.001) and in emotional resilience (+8.7, *p* = 0.002). Perceived stress decreased from 48% to 32% (p = 0.008), while clinical competency scores improved significantly (*p* < 0.001). Multivariable regression revealed that resilience increase was a strong predictor of improvements in clinical competency (R^2^ = 0.35, *p* < 0.001).

**Conclusion:**

This pragmatic, single−cohort curriculum was feasible to implement and was associated with improvements in cognitive engagement, emotional resilience, perceived stress, and clinical performance among Saudi medical interns, supporting further evaluation and potential broader adoption of such integrated programs in medical education.

## Introduction

Medical internship represents a pivotal, high-stakes transitional phase for newly graduated physicians, demanding the rapid consolidation of vast medical knowledge and the simultaneous cultivation of emotional resilience under extraordinary pressure (1). This critical period is universally recognized as one of the most challenging in medical training, characterized by long working hours, high patient acuity, and the immense responsibility of complex clinical decision-making (2). The relentless nature of these demands contributes significantly to a well-documented crisis of stress, burnout, and psychological distress among interns globally. Recent systematic reviews and meta-analyses report alarming burnout rates, with some studies indicating that over a third of all interns experience significant symptoms (3, 4).

The challenges faced by medical interns are complex and multifaceted. In addition to the cognitive demands of processing and applying an ever-growing body of medical knowledge, interns must also learn to handle emotionally charged situations involving patient suffering, ethical dilemmas, and medical uncertainties (5). These stressors are often intensified by systemic issues such as sleep deprivation, a heavy and unpredictable workload, and the inherent difficulties of shifting from the structured, theoretical environment of medical school to the chaotic, practical world of clinical care (6).

While this is a global issue, the context of Saudi Arabia and the broader Middle East and North Africa (MENA) region presents unique cultural and systemic factors. Although the prevalence of burnout is comparable to global figures, a significant knowledge gap exists regarding the development and evaluation of integrated, evidence-based curricula specifically tailored to this cultural and clinical context (1, 7). This highlights a critical need for innovative educational strategies that can support interns and optimize their learning while preserving their wellbeing.

### Theoretical framework: a dual-pillar approach to intern support

To address these multifaceted challenges, this study is grounded in a robust dual theoretical framework that integrates behavioral neuroscience (neurodidactics) and educational psychology. This integrated model provides a comprehensive foundation for designing an intervention that simultaneously targets both the cognitive and emotional dimensions of medical internship, moving beyond simplistic, single-focus solutions.

#### Pillar 1: cognitive enhancement through neurodidactics

This pillar provides the scientific foundation for our cognitive enhancement strategies by leveraging insights from neurodidactics, which bridges the gap between our understanding of neural mechanisms and the practice of education (8–10). The core principle guiding this pillar is Cognitive Load Theory (CLT), which posits that learning is hampered when the cognitive demands of a task exceed the finite capacity of an individual’s working memory (11). The internship environment, with its constant influx of new information and high-pressure tasks, is a prime setting for cognitive overload. Our curriculum directly addresses this by implementing evidence-based strategies designed to manage cognitive load and promote durable, long-term knowledge retention.

Retrieval practice and spaced repetition: Rather than passively reviewing information, our intervention actively employs retrieval practice, a technique proven to strengthen memory consolidation by forcing the brain to actively recall information (12). This is complemented by spaced repetition, which leverages the “spacing effect” to enhance long-term retention by distributing learning sessions over time. A meta-analytic review has confirmed that distributed practice significantly enhances classroom learning (13). Studies focusing on health professions have highlighted the power of these techniques in improving the retention and application of complex medical knowledge (14).

#### Pillar 2: emotional and affective regulation through educational psychology

This pillar informs the curriculum’s focus on emotional and affective regulation, drawing primarily from Self-Determination Theory (SDT). SDT is a major theory of human motivation that emphasizes the core psychological needs of autonomy (a sense of control over one’s actions), competence (a feeling of mastery and effectiveness), and relatedness (a sense of belonging and connection) as crucial drivers of well-being and intrinsic motivation (15). The hierarchical and high-stress nature of medical internships often threatens these basic needs.

Mindfulness and cognitive reappraisal: To bolster interns’ psychological resources, our curriculum incorporates mindfulness training and cognitive reappraisal exercises. Systematic reviews and meta-analyses have provided strong evidence that meditation and mindfulness-based programs can significantly reduce psychological stress, anxiety, and burnout in various populations, making them highly applicable to high-stress training environments, such as medical internships (16). Cognitive reappraisal, a technique for reframing stressful situations, further equips interns with the tools to manage their emotional responses effectively.

The rationale for this study is built on a critical observation: while the individual efficacy of cognitive learning strategies and emotional regulation techniques is well-established, there is a significant knowledge gap concerning the synergistic impact of an integrated curriculum that combines both. Most interventions target either cognitive skills or wellbeing in isolation. We argue that this siloed approach is fundamentally misaligned with the reality of medical practice, where cognitive and emotional processes are deeply intertwined. A physician’s ability to accurately diagnose a complex case (a cognitive task) is directly impacted by their ability to manage the stress of a high-stakes situation (an emotional task) (17).

Therefore, this study was designed to address this gap by developing and evaluating a holistic, integrated curriculum. We hypothesize that by simultaneously supporting both cognitive and emotional domains, the benefits will be synergistic, leading to greater improvements in clinical performance and professional resilience than either approach could achieve alone. Furthermore, by implementing and evaluating this program in the specific context of Saudi Arabia, we aim to generate much-needed, culturally relevant data that can inform the future of medical education in the MENA region. This study meets the need for evidence-based interventions in medical internship training by evaluating the aforementioned integrated curriculum. The primary objective was to assess the impact of this program on cognitive engagement, emotional resilience, perceived stress, and clinical performance among medical interns in Al-Madina Al-Monawara, Saudi Arabia. The secondary objective included assessing pre- and post-intervention changes in these metrics, comparing clinical competency scores, and exploring the link between changes in resilience and competency after the curriculum.

## Materials and methods

### Study design and setting

This study employed a pragmatic, quasi-experimental, single-cohort longitudinal design with pre- and post-intervention assessments, and focus groups were conducted to capture interns’ qualitative feedback regarding feasibility and perceived impact.

This design was selected to evaluate the effectiveness of the integrated curriculum in the real-world, high-pressure context of a medical internship, where a non-intervention control group was deemed ethically and logistically unfeasible (1, 2). The study was conducted over 6 months at three teaching hospitals in Al-Madina Al-Monawara, Kingdom of Saudi Arabia (KSA), from September to February 2024–2025.

### Participants and recruitment

A purposive sampling strategy was used. The total number of medical interns starting their rotational year at the participating hospitals was 145. From this cohort, 105 interns were deemed eligible to participate, and have all received an invitational email with a brief presentation during their internship orientation outlining the study. A total of 40 interns were excluded due to advanced training year timing that was incompatible with the study’s 6-month duration.

Interested interns received an information sheet and were allowed to ask questions in person or via email. Those who agreed to participate signed written informed consent before any baseline data were collected. A total of 100 interns provided informed consent, yielding a 95.2% response rate among those invited. All 100 consenting interns completed the 6-month implementation period, and no dropouts were recorded, resulting in 100% retention in the final analysis.

The inclusion criteria were to be a registered medical intern at one of the participating teaching hospitals, to be able to attend most scheduled sessions, and to be willing to complete pre- and post-intervention assessments. Interns who transferred to other institutions, took prolonged leave exceeding 4 weeks, or declined to participate were excluded. Participants served as their own controls in the pre–post design; a separate non-intervention control group was not included because withholding a potentially beneficial resilience-enhancing intervention from a subset of interns within the same cohort was deemed ethically and logistically inappropriate in this real-world setting.

### The integrated curriculum: intervention overview

The curriculum was systematically developed following the five phases of the ADDIE model (Analysis, Design, Development, Implementation, and Evaluation) to ensure a structured and evidence-based approach. The process began with a comprehensive Analysis of the main challenges and stressors faced by medical interns, drawing from an extensive literature review. This was followed by the Design phase, where, in consultation with subject-matter experts in medical education, neuroscience, and psychology, the curriculum structure was designed, and evidence-based strategies were selected. The topics were chosen to build foundational cognitive and emotional regulation skills. In the Development phase, a series of co-developed modules was created, integrating strategies in cognition, self-regulation, resilience, and communication. Cognitive and emotional components were deliberately co-delivered to reinforce their reciprocal effects. The modules were then pilot-tested and Implemented across rotations. Finally, the curriculum was Evaluated through pre-post assessments and focus groups to support ongoing refinement (18).

### Training implementation across medical rotations

**FIGURE 1:**
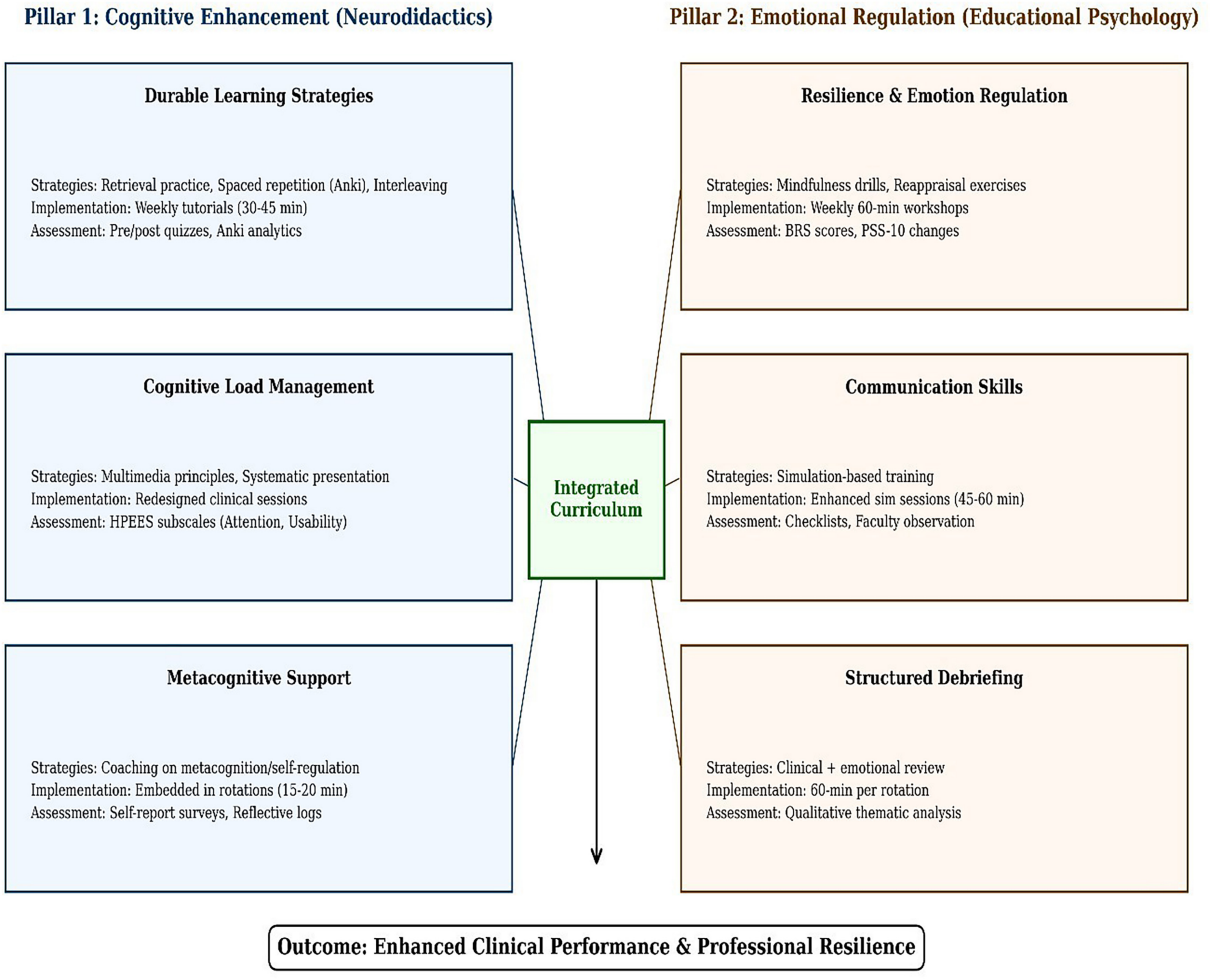
Integrated behavioral neuroscience and educational psychology curriculum. Schematic overview of modules embedded across rotations.

The integrated curriculum was implemented across the standard medical internship rotations, including Internal Medicine, Surgery, Pediatrics, Obstetrics and Gynecology, Emergency Medicine, and Family Medicine. Rather than creating separate modules, neuroscience and educational psychology principles were embedded in the existing rotation structure through enhanced teaching modalities. In each rotation, interns participated in modified tutorials that incorporated retrieval practice and spaced repetition using Anki flashcard software to consolidate their medical knowledge. Clinical teaching sessions were redesigned using multimedia principles of cognitive load to optimize information processing and retention. Simulation-based training sessions were enhanced with communication skills and team-based learning sessions. Additionally, weekly 60-min mindfulness and stress management workshops were conducted throughout all rotations to build emotional resilience. Each rotation also included weekly structured 60-min debriefing sessions that combined clinical learning with emotional regulation techniques, allowing interns to process challenging patient encounters while developing professional resilience (Figure 1).

### Data collection and outcome assessment

To assess the effectiveness of the curriculum, data were collected at baseline (before the intervention, represented as control) and at the end of the 6-month program. The primary outcome assessments were as follows:

1. Cognitive engagement: The Health Professions Education Engagement Scale (HPEES), a validated Arabic-language cognitive engagement survey instrument adapted for medical education contexts, was used to assess cognitive engagement (19). The HPEES consists of 20 items scored on a five-point Likert scale (1 = strongly disagree, 5 = strongly agree) and includes four subscales: focused attention, perceived usability, novelty and involvement, and artistic appeal. Total scores range from 20 to 100, with higher scores indicating greater engagement. The instrument has demonstrated good internal consistency (Cronbach’s α = 0.86) in health professions education contexts.
2. Emotional resilience: The Arabic version of the Brief Resilience Scale (BRS), validated for Arabic-speaking populations, was used to assess emotional resilience (20).
3. Perceived stress: The Arabic-validated Perceived Stress Scale (PSS-10) was used to assess perceived stress. This scale has demonstrated good reliability and validity in Arabic-speaking populations. PSS-10 total scores (range 0–40) were categorized according to commonly used interpretive ranges: low (0–13), moderate (14–26), and high (27–40). “High perceived stress” was defined as PSS-10 ≥ 27 (21).
4. Clinical performance: Rated by faculty members using the mini-Clinical Evaluation Exercise (mini-CEX), a widely used and validated tool for assessing clinical skills in the workplace (22). All faculty members who conducted the mini-CEX evaluations received standardized training on the assessment tool and scoring rubric to ensure inter-rater reliability and minimize assessment bias. To assess scoring consistency, a subset of 25 encounters was independently rated by two faculty members. Inter-rater reliability for the global mini-CEX score was good, with an intraclass correlation coefficient (two-way random effects, absolute agreement) of 0.82.

#### Qualitative focus groups

After completing the six-month curriculum, eight focus groups with 68 interns (aprox. 8 per group). Focus groups explored feasibility, acceptability, and perceived impact. Each 45–60-min session was audio-recorded, transcribed verbatim, and analyzed using inductive thematic analysis by two independent coders; discrepancies were resolved through discussion or consultation with a third reviewer.

### Data management and analysis

Quantitative data were entered into SPSS version 28.0 for analysis. Paired-samples *t*-tests compared pre- and post-intervention scores for continuous variables. Perceived stress (PSS-10) was analyzed as a categorical outcome (based on a predefined cutoff), and the pre–post change in the proportion of interns meeting the “high perceived stress” criterion was tested using McNemar’s test for paired categorical data. To account for baseline differences and isolate the unique contribution of changes in resilience, we also conducted multivariable linear regression analyses. Change in clinical competency (Δ mini-CEX score) was the dependent variable, with changes in emotional resilience (Δ BRS), baseline mini-CEX, baseline resilience, gender, and age as independent variables. Variance inflation factors were examined to exclude problematic multicollinearity. Assumptions of linearity, homoscedasticity, and normality of residuals were checked using residual plots and Shapiro–Wilk tests; no serious violations were identified. A two-sided *p*-value < 0.05 was considered statistically significant. Exploratory subgroup analyses examined whether the curriculum’s impact differed by gender and baseline stress level (high vs. low/moderate PSS-10). Interaction terms (e.g., Δ resilience × gender) were added to regression models, and stratified analyses were performed where appropriate.

### Trustworthiness and reflexivity

The research team acknowledged its dual role as developers and researchers. To mitigate bias, an independent team member conducted quantitative analysis, and findings were triangulated across data sources.

### Ethical considerations

The study was conducted in full accordance with the ethical principles for medical research involving human subjects. We obtained approval from the Institutional Review Board of the participating institutions, MFOM IRB HIST1-6, and all interns provided written informed consent before enrollment. Privacy and confidentiality were protected throughout the research process.

## Results

Of the 105 invited medical interns, 100 (95.2%) completed the full 6-month curriculum. The implementation of this integrated educational program has resulted in statistically significant improvements across all primary and secondary outcome measures. The key findings are presented below.

### Cognitive engagement, emotional resilience, and perceived stress

**FIGURE 2:**
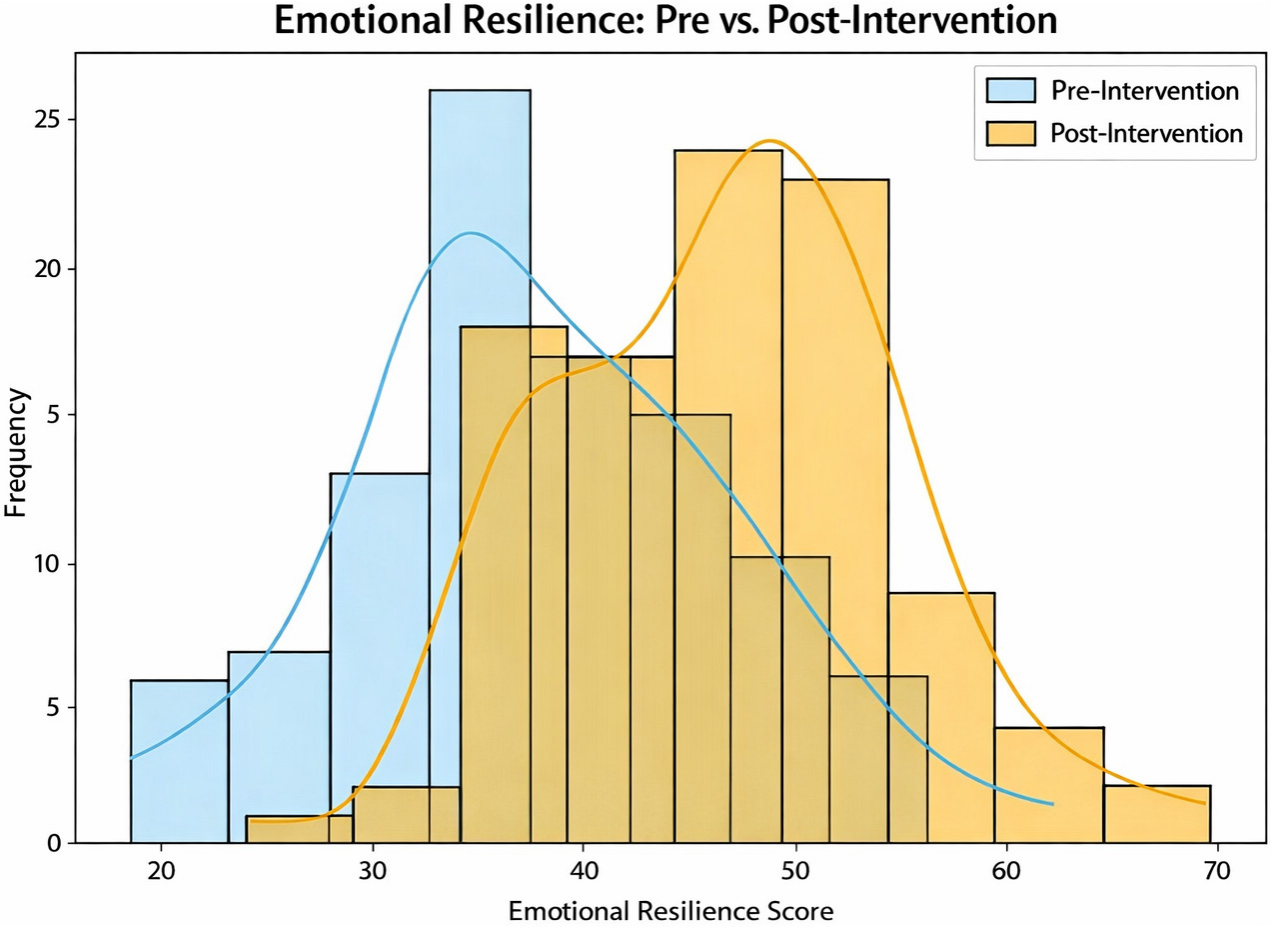
Distribution of emotional resilience scores (Brief Resilience Scale, BRS) among medical interns before and after the 6-month integrated curriculum (*N* = 100). Pre-intervention distribution (blue) shows lower resilience scores; post-intervention distribution (orange) demonstrates a significant rightward shift reflecting improved resilience (mean +8.7 points, 95% CI 5.5–11.9, *p* = 0.002).

As detailed in Table 1, after the 6-month integrated curriculum, participants showed significant improvement in cognitive engagement. Mean cognitive engagement scores increased from 45.0 ± 10.0 at baseline to 57.4 ± 9.0 after the intervention, representing a mean change of +12.4 points (95% CI 10.1 to 14.7; *p* < 0.001). A parallel improvement in emotional resilience was also observed, with mean scores rising from 38.0 ± 8.5 to 46.7 ± 8.0 after the intervention (Δ + 8.7; 95% CI 5.5 to 11.9; *p* = 0.002) (Figure 2).

**TABLE 1:** Pre- and post-intervention outcomes for cognitive engagement, emotional resilience, perceived stress, and clinical competency (*N* = 100).

Outcome Variable	Pre-intervention (Mean ± SD)	Post-intervention (Mean ± SD)	Mean Change [95% CI]	*p*-value
Cognitive engagement	45.0 ± 10.0	57.4 ± 9.0	+12.4 (10.1, 14.7)	< 0.001
Emotional resilience	38.0 ± 8.5	46.7 ± 8.0	+8.7 (5.5, 11.9)	0.002
Perceived stress (% high)*	48%	32%	−16% (−23%, −9%)	0.008
Clinical competency	70.0 ± 12.0	77.5 ± 11.0	+ 7.5 (5.0, 10.0)	<0.001

*Perceived stress (%), proportion with PSS-10 ≥ 27 (high perceived stress).

**FIGURE 3:**
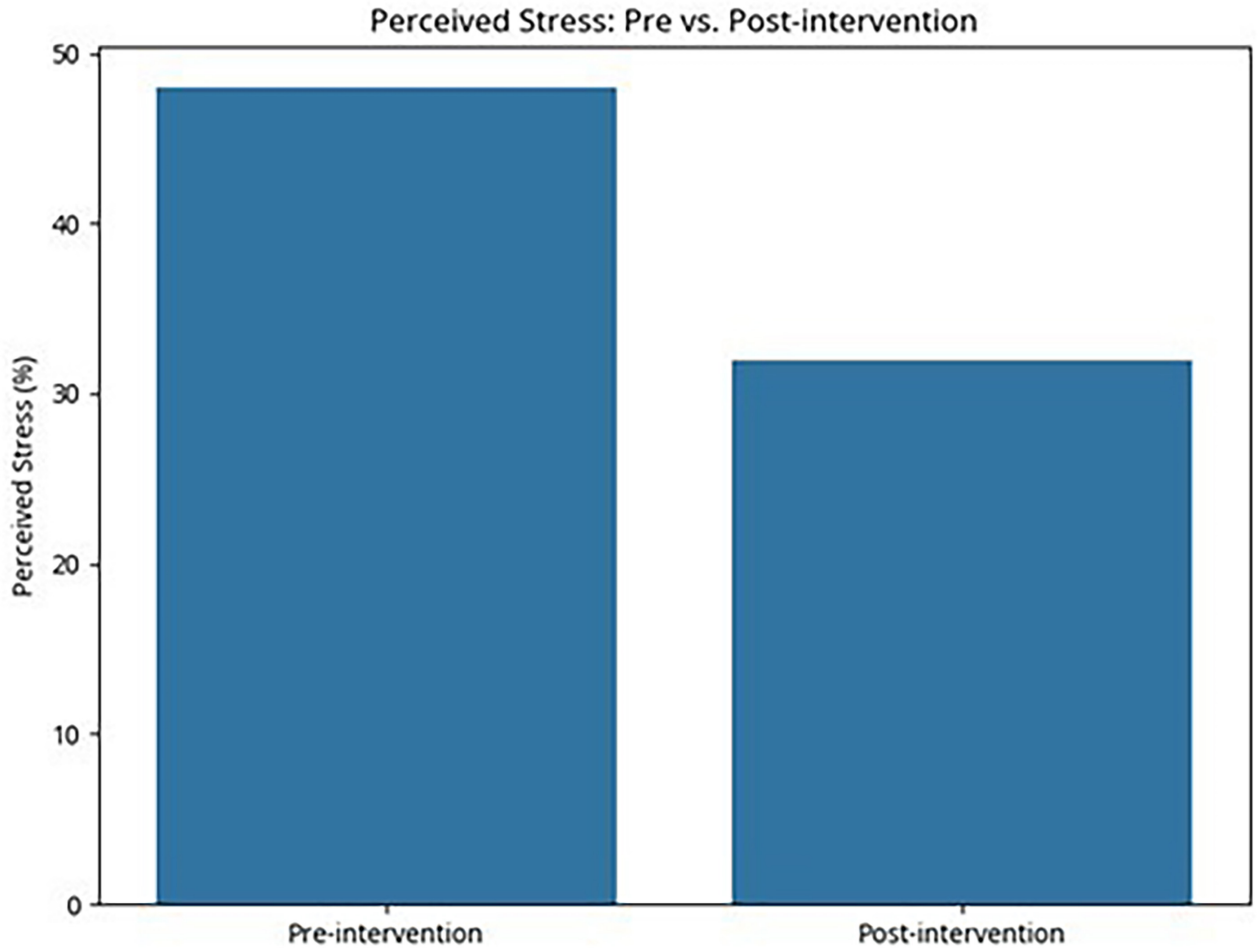
Proportion of medical interns reporting high perceived stress (PSS-10 ≥ 27) before and after the 6-month integrated behavioral neuroscience and educational psychology curriculum intervention (*N* = 100). The prevalence of high perceived stress decreased significantly from 48% pre-intervention to 32% post-intervention (absolute reduction 16%, 95% CI -23% to -9%, *p* = 0.008, McNemar’s test).

In contrast to these increases, perceived stress decreased. The proportion of interns meeting the criterion for high perceived stress decreased from 48% at baseline to 32% post-intervention, representing an absolute reduction of −16% (95% CI −23% to −9%; *p* = 0.008). These categories were used as interpretive guidelines rather than diagnostic thresholds (Figure 3).

### Clinical competency and the role of resilience

In addition to these psychological and emotional benefits, the curriculum intervention also led to a tangible improvement in the interns’ clinical skills, as assessed by faculty-rated mini-CEX. Clinical competency improved significantly from 70.0 ± 12.0 at baseline to 77.5 ± 11.0 post- intervention (mean change + 7.5, 95% CI 5.0 to 10.0; *p* < 0.001).

**FIGURE 4:**
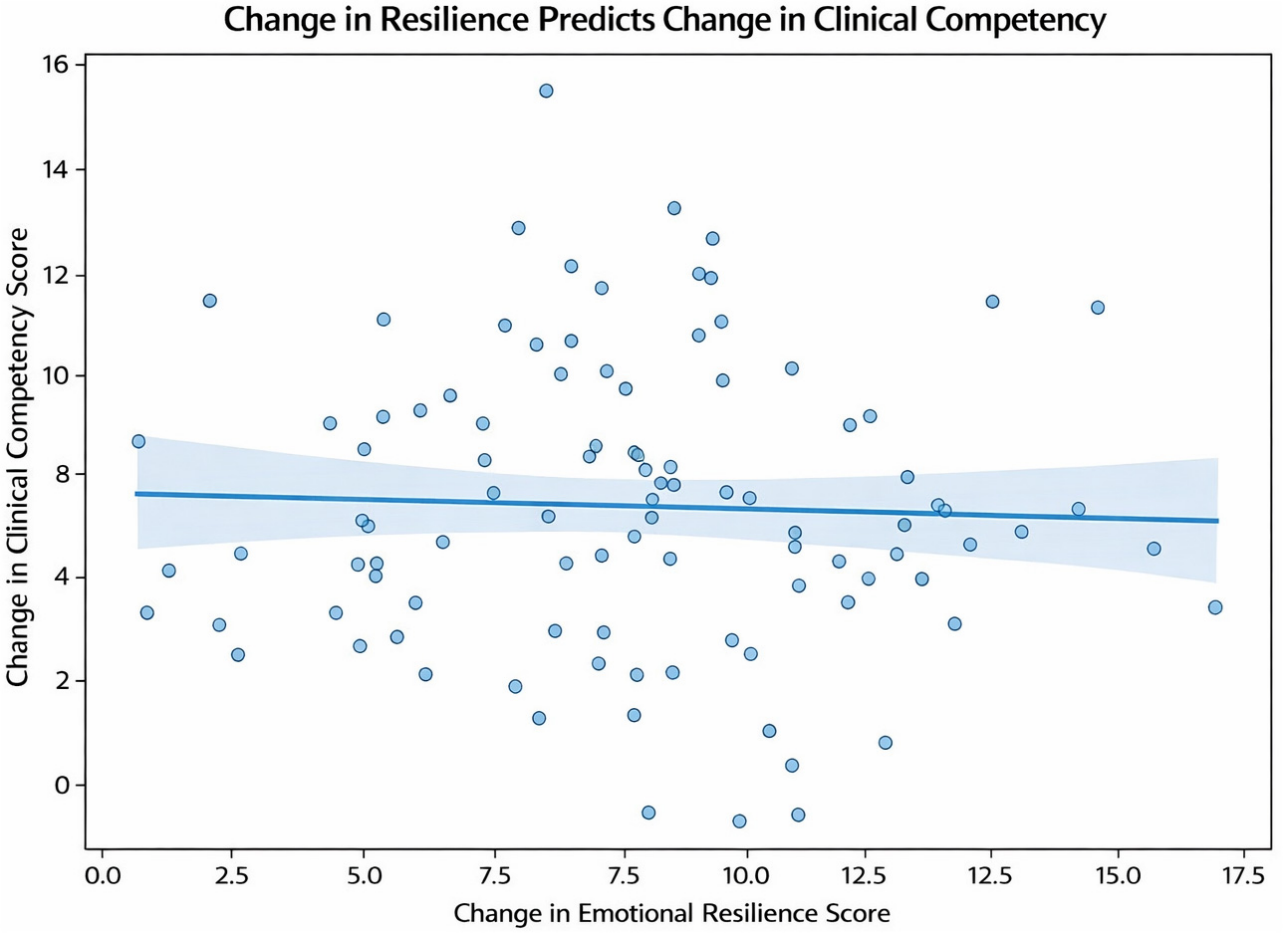
Change in emotional resilience (ΔBRS) predicts change in clinical competency (Δmini-CEX) among medical interns (*N* = 100). Each point represents one intern; the regression line shows a significant positive association (R^2^ = 0.35, *p* < 0.001; standardized β = 0.47 after adjustment for baseline scores).

To examine whether improvements in emotional functioning were associated with gains in performance, we tested the predictive role of resilience using regression analysis. Increases in emotional resilience significantly predicted improvements in clinical competency, with resilience gains accounting for 35% of the variance in changes in clinical competency scores (R^2^ = 0.35, *p* < 0.001). This proportion of explained variance indicates that additional unmeasured factors also contribute to clinical performance. In a multivariable model adjusting for baseline mini-CEX scores, baseline resilience, gender, and age, change in emotional resilience remained an independent predictor of improvement in clinical competency (standardized β = 0.47, *p* < 0.001). The full model accounted for 42% of the variance in Δ mini-CEX (adjusted R^2^ = 0.42), indicating that resilience gains contributed substantially to performance improvement even after controlling for key covariates. This strong, positive association highlights the crucial role of emotional regulation in the development of clinical skills (Figure 4).

#### Qualitative focus groups

Qualitative feedback strongly supported the feasibility and perceived educational value of the integrated curriculum, providing contextual insight into how cognitive and emotional improvements may translate into gains in clinical competency.

Key themes that emerged included:

1. Enhanced cognitive clarity and structured learning. Most interns (*n* = 54; 79%) reported that integrating spaced repetition flashcards (Anki) and retrieval practice into ward rotations created “more organized and memorable” learning experiences compared with traditional teaching. Representative quote: “The flash cards made studying less overwhelming and helped me recall information when I needed it most.”

2. Emotional resilience and stress management. Sixty-two interns (91%) reported that mindfulness and emotion regulation workshops offered practical, immediately applicable strategies for managing stress. Particularly valued were debriefing sessions that combined clinical learning with reflection on emotional responses. Quote: “After difficult cases, the debriefing sessions helped me process what happened and learn from it without feeling so alone.”

3. Improved team communication. A total of 51 interns (75%) reported that simulation-based communication training led to more confident, effective interactions with senior staff and peers. Quote: “The simulations gave me a safe space to practice assertiveness in communication, which I carried into my actual patient encounters.”

4. Acceptability and feasibility. All interns reported that the 60-min weekly workshops and weekly debriefing sessions were manageable within the demanding internship schedule, though 38 interns (56%) suggested that weekend sessions or flexible scheduling would improve access during particularly busy rotations.

5. Relevance to local context. Thirty-four interns (50%) explicitly commented that the curriculum addressed challenges specific to the hierarchical nature of training in Saudi hospitals, where speaking up or disclosing difficulty is culturally challenging. Quote: “This program taught me that asking for help and managing my stress are signs of strength and professionalism, not weakness.”

#### Exploratory subgroup analyses

Exploratory analyses suggested that the curriculum’s benefits were broadly similar across demographic groups. Both male and female interns showed significant pre–post improvements in resilience, stress, and clinical competency, with no statistically significant interaction between gender and resilience gains in predicting Δ mini CEX (interaction *p* = 0.41). Likewise, interns who entered the program with high baseline perceived stress (PSS-10 ≥ 27) showed clinical competency gains comparable in magnitude to those with low or moderate baseline stress, despite the high-stress group exhibiting slightly larger reductions in PSS-10 scores. Given the sample size, this subgroup findings should be interpreted cautiously and primarily as hypothesis-generating.

## Discussion

The results of this study provide compelling evidence that an integrated curriculum grounded in behavioral neuroscience and educational psychology significantly enhances the medical interns’ training experience. The observed improvements in cognitive engagement, emotional resilience, and clinical competency, coupled with a significant reduction in perceived stress, align with the broader literature on cognitive and affective learning and demonstrate the practical value of evidence-based educational interventions in medical training.

The significant improvements observed in this study can be directly interpreted through the lens of our dual theoretical framework. The gains in cognitive engagement and clinical competency are consistent with the principles of Cognitive Load Theory (23). By implementing strategies such as retrieval practice and spaced repetition, the curriculum was designed to manage the interns’ intrinsic cognitive load, thereby freeing up working memory resources for the complex task of clinical reasoning. This allowed for the development of more robust mental schemas, leading to the observed improvements in performance. Concurrently, the marked increase in emotional resilience and reduction in perceived stress align with Self-Determination Theory (15). The curriculum’s focus on mindfulness, supportive debriefing, and communication skills directly addressed the interns’ basic psychological needs for competence, autonomy, and relatedness. By fostering a learning environment that supported these needs, intervention likely enhanced intrinsic motivation and provided the psychological stability required to engage deeply with the demanding clinical work. Therefore, it is the synergistic application of both cognitive and psychological principles that likely explains the holistic and meaningful improvements seen in our cohort.

The significant gains in cognitive engagement are directly attributable to the systematic application of retrieval practice and spaced repetition. These results are consistent with previous work demonstrating the efficacy of these techniques across a range of instructional settings (15, 24–26) and support the principles of neurodidactics, which emphasize that learning is a dynamic process that can be modified through evidence-based interventions (8). Similarly, the notable improvements in emotional resilience and the corresponding decrease in stress levels are consistent with the growing body of evidence supporting mindfulness and emotion regulation training in high-stress professions (16, 27–29).

A key contribution of this study is the demonstration of a strong predictive relationship between increases in emotional resilience and improvements in clinical competency (R^2^ = 0.35). This relationship is consistent with theoretical models linking emotional regulation and resilience to executive functioning, working memory, and sustained attention in high-stakes environments. Interns who are better able to recover from setbacks, manage anxiety, and maintain psychological flexibility may be more capable of fully engaging in clinical encounters, processing complex information, and seeking feedback rather than withdrawing under pressure. Beyond statistical significance, the magnitude of change in this study is meaningful both clinically and educationally. An average increase of 7.5 points in mini-CEX scores corresponds to about two-thirds of a standard deviation, indicating more consistent performance in data gathering, clinical reasoning, and communication during routine encounters. Additionally, a 16% absolute reduction in interns reporting high perceived stress suggests that many trainees moved from stress levels linked to higher risks of burnout and errors to more manageable levels. The high retention (100%) and debriefing fidelity (ICC = 0.82) further support curriculum scalability in high-stakes postgraduate training (30).

These findings are particularly relevant within the context of Saudi Arabia’s healthcare education landscape. Unlike some Western models that have long emphasized individual autonomy, medical training in the MENA region often occurs within more hierarchical clinical environments where cultural expectations can make it challenging for junior trainees to speak up or express vulnerability. Our results, demonstrating the feasibility and effectiveness of a curriculum that builds psychological safety and resilience, extend prior work on neuroscience-based interventions by showing their applicability in resource-constrained Middle Eastern settings (31). While other studies have shown the benefits of mindfulness and cognitive training in Western contexts, our study is one of the first to demonstrate the successful implementation of an integrated curriculum in the MENA region, providing a culturally relevant model for other similar healthcare systems in the area. The practical implications are noteworthy, as a curriculum that explicitly focuses on cognitive engagement, emotional resilience, and communication skills is particularly advantageous in supporting Saudi interns as they navigate these pressures. This locally developed, theory-based program could serve as a culturally relevant model for other medical education systems in the region facing similar challenges.

The scalability and cost-effectiveness of the interventions, particularly the use of Anki software for spaced repetition, make this approach feasible for implementation throughout diverse medical education settings. This is particularly relevant for medical education systems in developing countries and the MENA region, where resource constraints may limit access to expensive educational technologies. Our study provides a model for an evidence-based, low-cost, and effective curriculum that can be adapted to address the specific needs of medical trainees in various contexts.

## Limitations

While the outcomes of this study are promising, it is important to acknowledge its limitations to ensure a balanced interpretation of the findings. First, the single-site design, confined to teaching hospitals in Al-Madina Al-Monawara, may limit the generalizability of the results to other healthcare systems or cultural contexts. Second, the quasi-experimental, single-cohort longitudinal design with pre- and post-intervention assessments, while pragmatic for a real-world setting and defensible given the ethical aspects of withholding a beneficial intervention, cannot definitively attribute the observed improvements solely to our intervention, as other factors (e.g., secular trends, maturation effects, concurrent institutional changes) may have influenced the outcomes. This is an acknowledged limitation of the design. Subsequent studies should employ multicenter randomized controlled trials to strengthen causal inference. Third, the purposive sampling strategy may introduce recruitment bias. Fourth, several of our outcome measures relied on self-report surveys, which may be subject to social desirability bias. The assessment of clinical competency, although conducted by trained faculty, remains somewhat subjective. Fifth, as developers of the curriculum, our role as researchers introduces the potential for bias; we have attempted to mitigate this through the reflexive measures described. Finally, this study did not assess the long-term effects of the intervention. It is highly recommended to conduct subsequent studies that tackle these limitations by employing multicenter randomized controlled trial designs, including objective performance measures and incorporating long-term follow-up to assess the sustainability of the observed benefits.

## Data Availability

The original contributions presented in this study are included in this article/supplementary material, further inquiries can be directed to the corresponding authors.
